# Trachoma: the beginning of the end?

**Published:** 2012

**Authors:** Danny Haddad

**Affiliations:** Director: International Trachoma Initiative www.trachoma.org

Blinding trachoma, one of the oldest known infectious eye diseases, may be facing its end. Formerly the world's leading cause of preventable blindness, trachoma has brought physical suffering and economic devastation to tens of millions of people, mostly women and children in poorer countries. As a result of development and targeted interventions, however, nine countries with trachoma have already reported achieving elimination targets, and more than 80 per cent of the burden of active trachoma is now concentrated in 14 countries.

But this is not enough. We want to ensure that trachoma is eliminated worldwide by 2020, and that the 4.6 million people suffering from trichiasis receive the sight saving surgery they need.

The World Health Organization (WHO)-endorsed SAFE strategy (Surgery, Antibiotics, Facial cleanliness and Environmental improvement) provides the framework whereby trachoma can be eliminated. But success depends on having in place everything we need to carry out all four elements of the strategy.

To help us get there, the International Trachoma Initiative has been working with partners in the International Coalition of Trachoma Control to create the global strategic plan called 2020 INSight. This plan looks at where we are, where we want to go, how we get there, and the cost and impact of eliminating blinding trachoma.

Scaling up public health interventions described in the SAFE strategy, including antibiotic treatment with Zithromax® (donated by Pfizer Inc.) and improved access to water and sanitation, are the most crucial elements of the plan. 2020 INSight also provides direction on what else is needed to reach this ambitious goal: country leadership, international coordination, logistical and planning support, and adequate funding.

All the elements of the plan must be in place by 2015 if we are to eliminate the disease by 2020. Delayed action will be costly. There is the human cost of vision loss or blindness that leads to loss of social status, stigmatisation, and exclusion from society. There is also the economic burden of trachoma on the lives of individuals, families, and communities. Conservative estimates suggest that the annual loss of productivity due to trachoma is between US $3 billion and US $6 billion, and that eliminating trachoma in Africa will boost the continent's gross domestic product (GDP) by 20%–30%.

**Figure:**
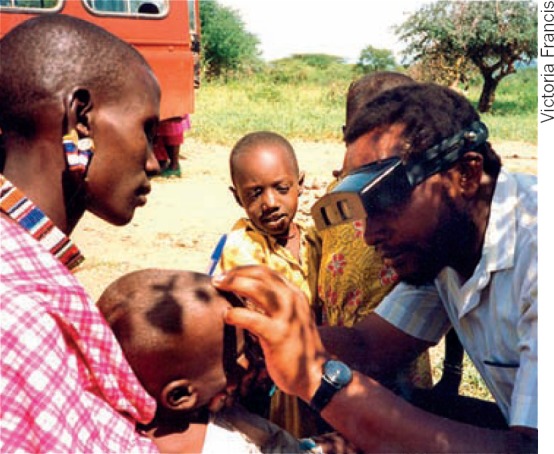
Examining a child for trachoma

Those of us involved in the fight against trachoma are hopeful. We have made much progress over the past 12 years. If the global community uses this new strategic plan to focus its efforts, attention, and funding, trachoma does not stand a chance.

Trachoma surgeons and community health workers around the world have a role to play. We invite you to contact us to learn more about what you can do. Please visit www.trachoma.org or read more about 2020 INSight at http://trachoma.org/global-strategy-2020-INSight

**Scaling up the SAFE strategy**

2020 INSight suggests the following:

**S (surgery)**

There are an estimated 4.6 million people who need surgery in the districts with confirmed trachoma. To clear this backlog would require the global community to scale up from 160,000 to more than 500,000 operations per year. Training trachoma surgeons is just the first step – there are many other challenges, including low productivity.

**A (antibiotics)**

More mass drug administration (MDA) programmes must be rolled out. The main barriers are getting the drugs to the target population. Co-distribution with other drug programmes can help.

**F (facial cleanliness)**

Behavioural change initiatives are needed in all districts, including about 500 new districts, but this remains difficult. Co-ordination with broader campaigns can be part of the solution (e.g., including face washing in national hand washing campaigns).

**E (environmental improvement)**

Access to clean water and latrines must be improved dramatically. There are still difficulties, but the trachoma community can push for more co-ordination and better information sharing with ministries and other partners focused on water and sanitation.

**EYE HEALTH HERO: Alemayehu Sisay**

**Figure:**
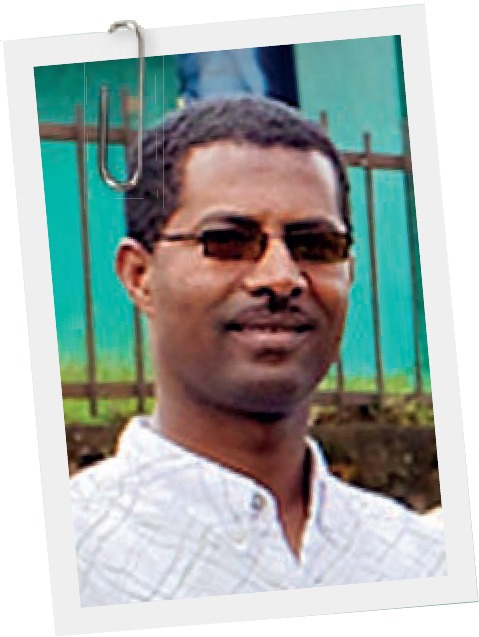

The international sight-saving organisation ORBIS has nominated programme manager and ophthalmologist Alemayehu Sisay as an Eye Health Hero for the International Agency for the Prevention of Blindness (IAPB) 9th General Assembly.

Dr Sisay, who works in southern Ethiopia, is a hands-on programme manager, travelling to rural areas and remote health points to assess projects, make recommendations, and assist with professional development of eye care professionals working to eliminate trachoma.

Driven by passion for the cause and an overriding sense of duty, Dr Sisay has been known to stop people in the street if he thinks that they have symptoms of trachoma. On his way to meetings or program assessments, it is not unusual for him to examine people and direct them to the nearest source of help.

The goal of ORBIS, Dr Sisay, and international partners, including the International Trachoma Initiative, is to eliminate trachoma globally by 2020 using the WHO-endorsed SAFE strategy.

As an Eye Health Hero, Dr Sisay will be able to attend the IAPB 9th General Assembly in Hyderabad, 17–20 September, where he will be able to meet the world's leading thinkers in prevention of blindness.

For more information, visit www.9ga.iapb.org

